# Confocal polarization tomography of dielectric nanocavities

**DOI:** 10.1515/nanoph-2024-0744

**Published:** 2025-04-23

**Authors:** Frederik Schröder, Martin P. van Exter, Meng Xiong, George Kountouris, Martijn Wubs, Philip T. Kristensen, Nicolas Stenger

**Affiliations:** Department of Electrical and Photonics Engineering, Technical University of Denmark, Ørsteds Plads 343, 2800 Kgs. Lyngby, Denmark; NanoPhoton – Center for Nanophotonics, Technical University of Denmark, Ørsteds Plads 345A, 2800 Kgs. Lyngby, Denmark; Huygens-Kamerlingh Onnes Laboratory, Leiden University, P.O. Box 9504, 2300 RA, Leiden, The Netherlands

**Keywords:** extreme dielectric confinement, fano lineshape, polarization tomography, confocal microscopy

## Abstract

We employ polarization tomography to characterize the modal properties of a dielectric nanocavity with sub-wavelength mode confinement. Our analysis of reflection spectra shows that the Fano-lineshape depends strongly on the polarization in a confocal configuration, and that the lineshape can be transformed into a Lorentzian-like peak for a certain polarization. For this polarization setting, the background is almost fully suppressed in a finite range of frequencies. This enables us to identify another resonance that has not yet been experimentally reported for these nanocavities. Lastly, we use symmetry-forbidden polarizations and show that, surprisingly, the modal resonance features of the system remain visible.

## 1 Introduction

Optical nanocavities, such as photonic crystal cavities [1], [2] and nanobeam cavities [3], play a crucial role in enhancing light–matter interactions [4] and are vital for applications ranging from efficient nanolasers [5], [6] to quantum technologies [7], [8], [9], [10]. Recent breakthroughs in dielectric cavity design have enabled confinement of electromagnetic fields on sub-wavelength scales [11], [12], [13], [14], [15], [16], [17]. In particular, cavities resulting from inverse design by topology optimization [18], [19], [20] have been realized in silicon [16] and indium phosphide [17] and have inspired further simplified designs [21]. Importantly, these extreme dielectric confinement (EDC) cavities are not limited by absorption in the material and can achieve quality factors several orders of magnitude higher than those of plasmonic structures [22], [23], [24]. The emergence of EDC cavities therefore represents a paradigm shift in device design, in which their strong field enhancements, typically achievable only with plasmonic systems but without the associated losses, open the door to unique light–matter interaction regimes.

To fully harness the potential of EDC cavities, it is essential to analyze their spatial and spectral properties in detail. Polarization-based tomography in a confocal microscope [25] is an effective technique for mode identification, which enables characterization of the associated resonance energies, polarization states, linewidths, and spatial distributions, even though the resolution is limited by the spot size of the focused light beam. This versatile method has been successfully applied to various photonic structures, including photonic crystals [26] and vertical-cavity surface-emitting lasers [27], [28]. Reflection spectra of EDC cavities typically have a Fano lineshape [15], [16], [17], resulting from interference of a spectrally narrow resonance with a slowly varying background [29], also observed in other types of cavities, like photonic crystal cavities [30], [31] and plasmonic resonators [32], [33]. This complicates the identification and quantification of resonance energies and quality factors, and understanding and exploiting this interference effect has attracted much interest [33], [34], [35], [36], [37], [38]. Several publications also consider the influence of polarization on the Fano lineshape [39], [40], [41], [42], [43], and theoretical studies have investigated polarization-dependent lineshape in photonic crystal cavities [44].

In this work, we present an adapted method of polarization tomography to explore the spectral, spatial, and polarization properties of topology optimization-inspired EDC cavities. Our approach goes beyond conventional cross-polarization configurations by incorporating variation in the polarization angles. We show that the reflection measurements in a confocal geometry depends crucially on the polarization, and we exploit this fact to demonstrate that the background can be completely eliminated over specific frequency ranges and for a certain elliptical polarization projection of the detected light. The lineshape transforms into a Lorentzian-like peak, and the approach enables the isolation of spectrally close resonances that are difficult to distinguish in standard configurations. In this way we identify an otherwise hidden feature in the experimental spectrum, which we are able to identify with numerical simulations.

This general method can be applied to other types of nanocavities and complements scattering-type scanning near-field optical microscopy measurements by providing precise information about cavity polarization properties while eliminating interference effects. We find that the method offers impressive detail in characterizing EDC cavities, which is crucial for optimizing light–matter interactions. This, in turn, enables design and modeling of future devices exploiting nonlinearities at the single-photon level for quantum information processing [45] and for the development of low-noise lasers for energy-efficient optical communications [46].

## 2 Polarization tomography of cavity resonances

Polarization tomography can be conveniently performed using reflection spectroscopy in a confocal geometry, in which focused input and output beams are used, respectively, to illuminate the sample and collect the scattered light. The superior spatial control of this setup enables a selective excitation of specific resonances and a tailored background suppression.

Following the ideas of coupled-mode theory for describing coupled cavity-waveguide systems [47], we take the illumination and collection to be described by the two-dimensional vector functions ${\vec{S}}_{\text{in}}\left(\omega \right)$ and ${\vec{S}}_{\text{out}}\left(\omega \right)$ . They relate to the electric field in the focal plane via $\vec{E}\left(x,y,\omega \right)=\sqrt{\frac{\epsilon_{0}}{2}}f_{\text{c}}\left(x,y\right)\vec{S}\left(\omega \right)$ , where *f*_c_(*x*, *y*) describes the electric-field distribution in the focal plane governed by the optical setup [48]. For most setups, *f*_c_(*x*, *y*) is a two-dimensional Gaussian function. Without loss of generality, we scale the vector functions so that $|{\vec{S}}_{\text{in}}\left(\omega \right){|}^{2}$ and $|{\vec{S}}_{\text{out}}\left(\omega \right){|}^{2}$ provide the delivered input and collected output power, respectively, and for a chosen focal point of the illumination and collection optics **r**_0_ = (*X*, *Y*, *Z*) we can relate them by use of a 2 × 2 reflection matrix *r*(*ω*) as

(1) $$\left(\begin{array}{c} S_{\text{out},x}\left(\omega \right)\\ S_{\text{out},y}\left(\omega \right) \end{array}\right)=\left(\begin{array}{cc} r_{xx}\left(\omega \right) & r_{xy}\left(\omega \right)\\ r_{yx}\left(\omega \right) & r_{yy}\left(\omega \right) \end{array}\right)\left(\begin{array}{c} S_{\text{in},x}\left(\omega \right)\\ S_{\text{in},y}\left(\omega \right) \end{array}\right).$$

In general, *r*(*ω*), and in turn ${\vec{S}}_{\text{out}}\left(\omega \right)$ , depend on the focal point position **r**_0_ with respect to the cavity, and we exploit this dependence to characterize the spatial properties of the detected signal in Section 4. If the sample has *x* and *y* mirror symmetry, and if the illumination and detection are on-axis, the off-diagonal elements of *r*(*ω*) vanish. Practical samples are never perfect, and small deviations from mirror symmetry in sample or optical alignment can create small but non-zero off-diagonal elements. Contrary to the off-diagonal elements, the on-diagonal coefficients can be relatively large and contain signatures of different resonances that typically couple primarily to *x*- or *y*-polarized input or output.

In optics, sharp spectral resonances are often situated on top of a broad spectral background and are visible as Fano profiles [49]. For a single resonance, the spectrum of the combined field can then be written as

(2) $${\vec{S}}_{\text{out}}\left(\omega \right)=\vec{b}\left(\omega \right)+\frac{\vec{a}}{1-i\left(\omega -\omega_{0}\right)/\gamma },$$

where $\vec{a}$ is a two-dimensional vector related to the field of a discrete mode that resonates at frequency *ω*_0_ with a damping rate *γ* and quality factor *Q* = *ω*_0_/(2*γ*). Mathematically, the discrete modes are known as quasinormal modes [50], [51], [52], [53] or resonant states [54], [55], [56], and are defined as solutions to the wave equation with suitable radiation conditions to model light propagating away from the resonator. The spectral background $\vec{b}\left(\omega \right)$ is typically slowly varying. The frequency dependence of $\vec{a}$ can be neglected since it is determined by the frequency dependence of the optical setup, which is expected to be constant in the spectral range of the cavity mode’s linewidth. The power spectrum produced by the field in Eq. (2) has the form of the previously-reported Fano lineshape [16], [30]:

(3) $$P\left(\omega \right)=A_{0}\left(\omega \right)+F_{0}\frac{{\left(q+\left(\omega -\omega_{0}\right)/\gamma \right)}^{2}}{1+{\left.\left(\omega -\omega_{0}\right)/\gamma \right)}^{2}},$$

where *F*_0_ is related to the amplitude, and *A*_0_(*ω*) denotes the offset spectrum, which determines the background via *A*_0_(*ω*) + *F*_0_. See Sec. S1 in the Supplementary Information [57] for an explicit calculation of the power spectrum in Eq. (3). *P*(*ω*) becomes a Lorentzian dip/peak for *q* → 0, ±*∞*, respectively.

**Figure 1:**
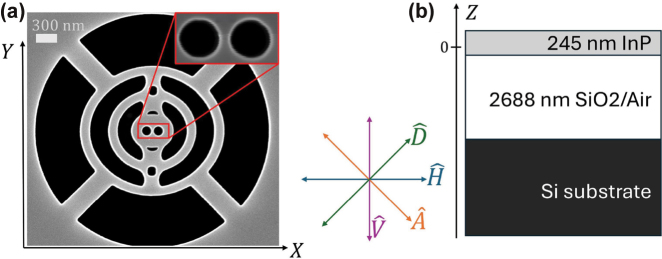
Sample geometry and polarization states. a) SEM image of a nominal equal cavity, defining the cartesian coordinates *X* and *Y*, as well as the linear polarizations $\hat{H}$ , $\hat{V}$ , $\hat{D}$ and $\hat{A}$ . The center of the cavity is taken as the origin of *X* and *Y*. b) Sketch of a cross-section of the sample. The SiO_2_ layer is etched under the cavity region, e.g. in the structured circular region in a) with a diameter of ≈ 3.5 µm.

The polarizations of the resonant contribution and of the background can be quite different. Whereas $\vec{a}$ is typically linearly polarized, the orientation of $\vec{b}\left(\omega \right)$ is generally more complicated. Typically, reflection measurements are performed in a cross-polarization configuration [30], [31], [36], [58] with the input and output polarizations oriented at −45° and 45° with respect to $\vec{a}$ , see the inset of Figure 1a. The polarization-projected background ${\tilde{b}}_{x}\left(\omega \right)-{\tilde{b}}_{y}\left(\omega \right)$ observed in this geometry disappears when ${\tilde{b}}_{x}\left(\omega \right)={\tilde{b}}_{y}\left(\omega \right)$ , i.e. when the background has no polarization preference. For the general case ${\tilde{b}}_{x}\left(\omega \right)\neq {\tilde{b}}_{y}\left(\omega \right)$ the spectral background can, in principle, also be removed by simply placing a quarter-wave plate in front of the analyzer and setting both components at convenient angles. This follows from the fact that $\vec{b}\left(\omega \right)$ has a well-defined polarization at any frequency *ω*. This suppression should be possible at any fixed frequency by using optimized polarization settings. We demonstrate this general background suppression in a finite range of frequencies in Section 4.

## 3 Sample and setup

We investigated an EDC cavity as the one depicted in Figure 1. This design [21], which results from a simplification of the cavity in Ref. [16], features an optical cavity mode with strong field confinement, as verified by scattering-type scanning near-field optical microscopy (s-SNOM). The mode of interest has a nominal resonance energy of (1.10834 ± 1 × 10^−5^) eV and has a nominal quality factor of 719 ± 3 along with an effective mode volume $\left(0.057\pm 0.003\right){\left(\lambda_{0}/n_{\text{InP}}\right)}^{3}$ as calculated in the center of the cavity. See Sections S6 and S7 of the Supplementary Information [57] for details of the s-SNOM measurements and the numerical simulations. The electric-field of the cavity modes must obey the two-fold mirror symmetry of the structure in the *XY* plane, and we find that the mode of interest is dominantly linearly-polarized along the $\hat{V}$ direction, see S7 in the Supplementary Information [57].

The cavity was fabricated in a 245 nm thick layer of indium phosphide (InP) on 2,688 nm silicon dioxide (SiO_2_) on a silicon (Si) substrate as described in [17]. The SiO_2_ layer served as a sacrificial layer, yielding a membranized cavity. In the experiments we performed spatial scans, where we moved the sample in the *X* − and *Y* − directions, as defined in Figure 1a. Moreover, we pursued reflection measurements for several linear polarizations, horizontal $\hat{H}$ , vertical $\hat{V}$ , diagonal $\hat{D}$ , and antidiagonal $\hat{A}$ , cf. Figure 1a.

**Figure 2:**
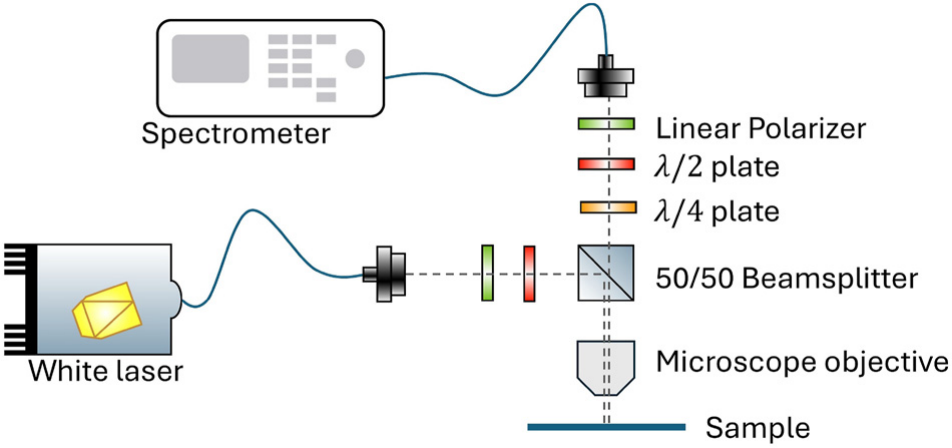
Sketch of the confocal reflection spectroscopy setup. The components are described in the main text.

The reflection measurements were carried out in a standard confocal geometry, as depicted in Figure 2. A supercontinuum laser (SuperK COMPACT, NKT Photonics) was used as a white light source in a spectral range from 450 nm to 2,400 nm. We used a long-pass filter with a cut-on wavelength of 1,100 nm to prevent saturation of the detector and minimize the fluorescence signal. A linear polarizer and a half-wave (*λ*/2) plate were used to control the polarization of the input light, which was focused on the sample with a 50 × microscope objective (LCPLN50XIR, Nikon, NA = 0.65). A 50/50 beamsplitter (BSW29R, Thorlabs) enabled illumination and detection through the same objective to measure in a reflection geometry. Another linear polarizer and *λ*/2 plate-combination ensured polarization control of the reflected beam. All waveplates were achromatic for broadband operation. For spatial filtering, we collected the reflected beam with a single-mode fiber in the detection path. The collection fiber was coupled to a spectrometer equipped with an indium gallium arsenide camera. All measurements were carried out with a 150 lines/mm grating. A quarter-wave (*λ*/4) was inserted in certain specific cases, but unless explicitly stated otherwise, the data presented were acquired without the *λ*/4 plate.

All acquired spectra have been normalized by a reference spectrum taken on a monocrystalline gold (Au) flake of high quality [59]. Additionally, we recorded spectra as a function of the *Z*-position. This way, the out-of-plane confinement of the focal spot can be determined to have a full-width half-maximum FWHM = (2.0 ± 0.1) μm at *E*_ph_ = 1.116 eV, see Sec. S2 of the Supplementary Information [57].

The transmission and reflection coefficients of the beamsplitter were polarization- and wavelength-dependent. We have determined these coefficients by comparing spectra measured under different polarization conditions and used this information to convert most of our data into reflectivity spectra, see also Sec. S3 of the Supplementary Information [57].

**Figure 3:**
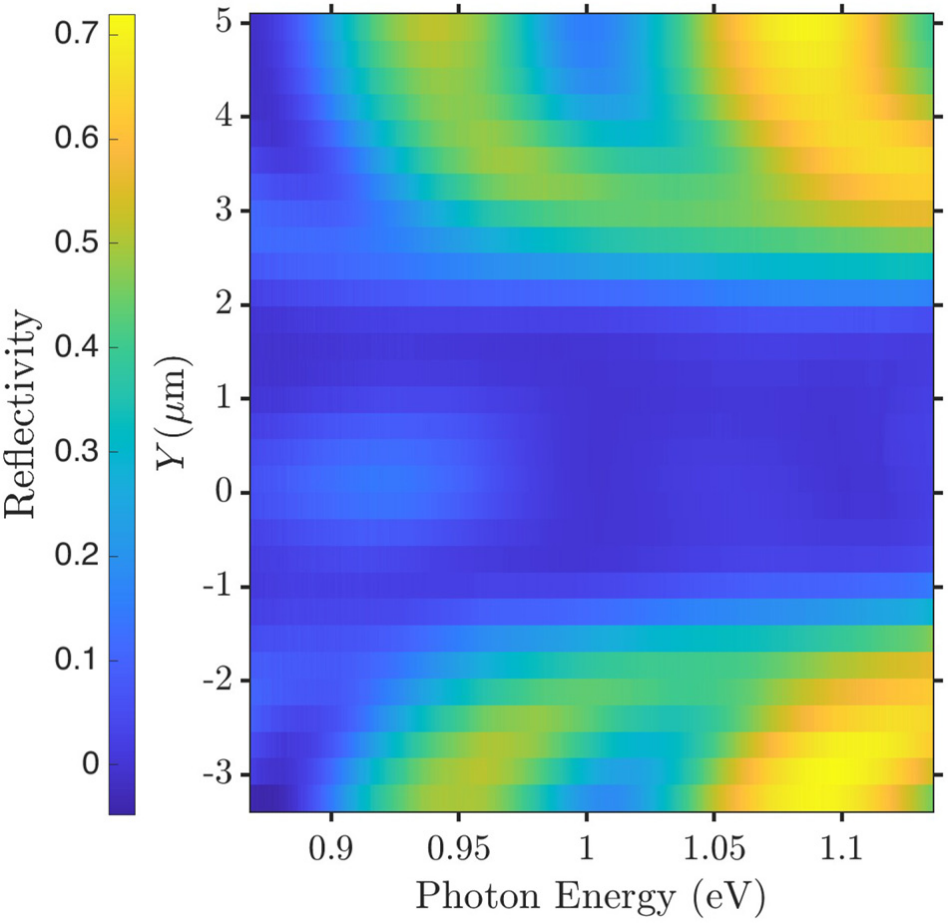
Reflection spectra of the cavity as a function of the *Y*-position at *X* = 0 in parallel polarization $\left({\hat{E}}_{\text{in}}={\hat{E}}_{\text{out}}=\hat{D}\right)$ .

To assess the lateral resolution of our setup, we recorded reflection spectra while moving the sample under the objective with the input- and output polarization parallel to each other. Figure 3 shows an example of a *Y*-scan with the input and output electric field-polarizations, ${\hat{E}}_{\text{in}}$ and ${\hat{E}}_{\text{out}}$ , both along the diagonal $\hat{D}$ . Clearly, the overall reflection is higher from the substrate than the cavity region, which can be explained by Fresnel reflection from the InP/SiO_2_ substrate. Notably, peaks are observed around 0.94 eV and 1.09 eV. Those are well-explained by Fabry–Pérot type resonances in the SiO_2_ layer sandwiched between two materials with higher refractive index. Indeed, with the resonance condition for a Fabry–Pérot cavity *mλ*_*m*_ = 2*n*_SiO2_ *d*, where *m* denotes the order of the resonance, *d* the thickness and *n*_SiO2_ the refractive index of the SiO_2_ layer, one calculates *E*_ph,m=6_ = 0.95 eV and *E*_ph,m=7_ = 1.09 eV.

The reflected signal reduced drastically when the beam was focussed in the void regions next to the outer rings of the cavity and increased slightly when the focal spot was in the center of the cavity close to *Y* = 0, cf. Figure 3 and Fig. S4.1 of the Supplementary Information [57]. We determined the spatial resolution of the setup by integrating the spectra and fitting the edges with an error function as expected from the convolution of a Gaussian resolution function and an edge function, see Fig. S4.1 of the Supplementary Information [57]. In this way, we found a FWHM for the lateral resolution of (1.0 ± 0.2) μm, which is close to the theoretical value of 0.87 μm that would correspond to a diffraction-limited spot at *E*_ph_ = 1.116 eV.

## 4 Results

**Figure 4:**
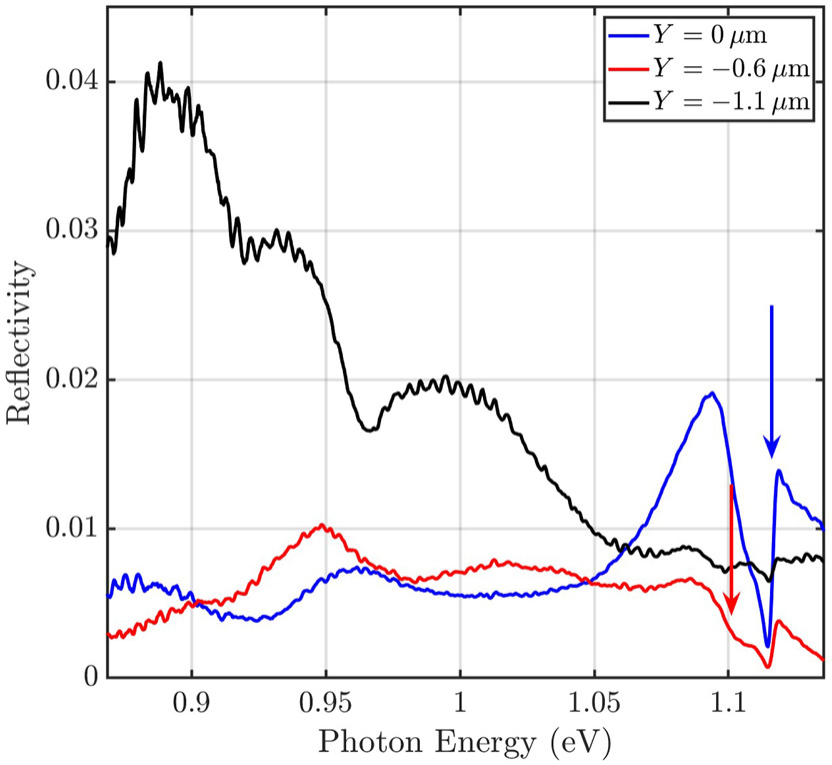
Spectra at different *Y* positions in the conventional cross-polarization configuration ${\hat{E}}_{\text{in}}=\hat{A}$ , ${\hat{E}}_{\text{out}}=\hat{D}$ . The blue and red arrows mark the resonances of the high-*Q* mode and of the low-*Q* mode deduced from fits, respectively (cf. Fig. A.1 and Fig. A.2).

**Figure A.1:**
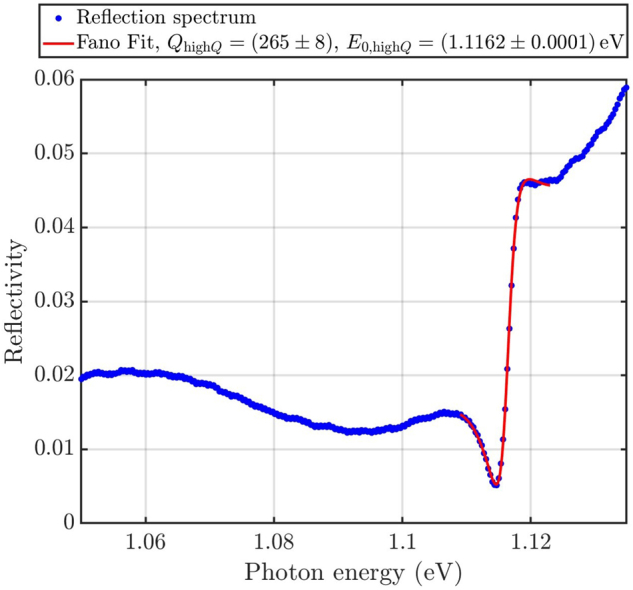
Reflection spectrum in the center of the cavity in parallel-polarization along the polarization of the high-*Q* mode ${\hat{E}}_{\text{in}}={\hat{E}}_{\text{out}}=\hat{V}$ . The high-*Q* mode has been fitted after Eq. (3).

**Figure A.2:**
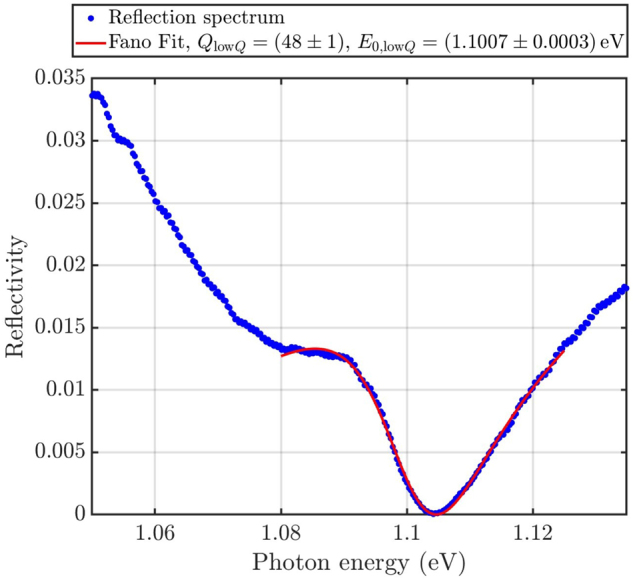
Reflection spectrum in the center of the cavity in parallel-polarization along the polarization of the low-*Q* mode ${\hat{E}}_{\text{in}}={\hat{E}}_{\text{out}}=\hat{H}$ . The low-*Q* mode has been fitted after Eq. (3).

Figure 4 shows spectra in the standard cross-polarization configuration ${\hat{E}}_{\text{in}}=\hat{A}$ , ${\hat{E}}_{\text{out}}=\hat{D}$ for different *Y* positions and for *X* = *Z* = 0. A previously reported cavity mode [21] is visible, marked by the blue arrow. As evident from a fit and from simulations (see Fig. A.1 and Fig. S7.1 in the Supplementary Information [57]), this mode has the highest quality factor. Henceforth, this mode is referred to as the high-*Q* mode. When the focus spot is moved by 0.6 μm from the center of the cavity, another mode becomes faintly visible at *E*_ph_ = 1.10 eV (see red arrow in Figure 4). We deduce a lower quality factor of this mode (see Fig. A.2 and Fig. S7.1 in the Supplementary Information [57]), which is why we label this mode as the low-*Q* mode. Finally, at about 0.9 μm to 1.2 μm away from the center, spectral features in a broad range between 0.87 eV and 1.04 eV become prominent, see also Fig. S5.1. This could be attributed to Fano-interferences, related to other eigenmodes of the membrane with lower resonance energies and Fabry–Pérot-type resonances forming in the air layer between the membrane and the silicon substrate. This assignment is supported by an eigenmode simulation of the membrane in Sec. S7 in the Supplemental Information, together with a simple model for Fabry–Pérot resonances, see below.

**Figure 5:**
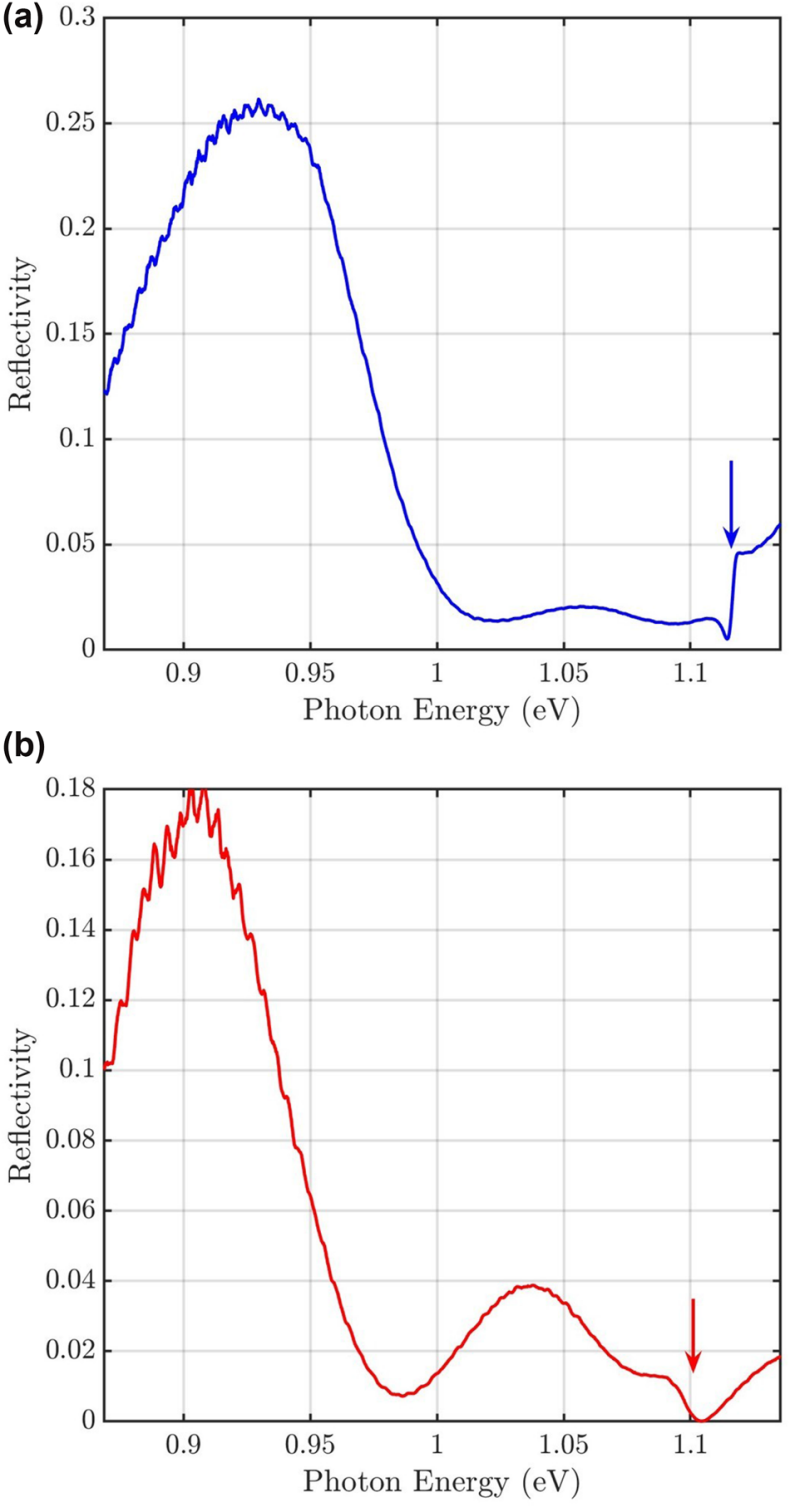
Reflection spectrum in the center of the cavity (*X* = *Y* = *Z* = 0), recorded in parallel polarization. a) ${\hat{E}}_{\text{in}}=\hat{V}$ , ${\hat{E}}_{\text{out}}=\hat{V}$ . The blue arrow marks the resonance energy of the high-*Q* mode deduced from a fit (cf. Fig. A.1). b) ${\hat{E}}_{\text{in}}=\hat{H}$ , ${\hat{E}}_{\text{out}}=\hat{H}$ . The red arrow marks the resonance energy of the low-*Q* mode deduced from a fit (cf. Fig. A.2).

The high-*Q* mode is dominantly polarized along the $\hat{V}$ direction (cf. Fig. S7.2) [21]. Therefore, we measure the reflection spectrum in a parallel configuration, where the input and output polarizations are aligned with the polarization of the high-*Q* mode. Figure 5a depicts a reflection spectrum for ${\hat{E}}_{\text{in}}=\hat{V}$ , ${\hat{E}}_{\text{out}}=\hat{V}$ . The high-*Q* mode, marked by the blue arrow, is clearly visible, while the low-*Q* mode is suppressed. Moreover, a peak around *E*_ph_ ≈ 0.93 eV can be observed, which is close to the value expected for a Fabry–Pérot mode *E*_ph,m=4_ = 0.92 eV forming in the air layer (*n*_Air_) between the cavity and the silicon substrate.

The reflection spectrum is fitted with Eq. (3) close to the resonance of the high-*Q* mode. For the sake of reducing the number of free-fitting parameters, *A*_0_(*E*_ph_) is assumed to be a linear function of *E*_ph_ in the vicinity of the resonance. We note that this effectively is a first-order Taylor approximation of the offset spectrum. The fit of the spectrum in the center of the EDC cavity for ${\hat{E}}_{\text{in}}=\hat{V}$ , ${\hat{E}}_{\text{out}}=\hat{V}$ yields *E*_0,high*Q*_ = *ℏω*_0,high*Q*_ = (1.1162 ± 0.0001) eV and *Q*_high*Q*_ = 265 ± 8, cf. Fig. A.1.

Measuring the reflection with ${\hat{E}}_{\text{in}}=\hat{H}$ , ${\hat{E}}_{\text{out}}=\hat{H}$ , we suppress the reflection of the high-*Q* mode, while the low-*Q* mode remains clearly visible (cf. Figure 5b). We conclude that the low-*Q* mode is predominantly polarized along the $\hat{H}$ direction. A fit with Eq. (3) of the low-*Q* mode yields *E*_0,low*Q*_ = (1.1007 ± 0.0003) eV and *Q*_low*Q*_ = 48 ± 1, see Fig. A.2. Consequently, the low-*Q* mode has a lower quality factor, lower resonance energy, and orthogonal polarization compared to the high-*Q* mode. An investigation of the spatial extent of those two modes, both with far-field reflection measurements in the confocal geometry as well as near-field measurements with a s-SNOM, can be found in Sec. S5 and S6 in the Supplementary Information [57]. Comparing the spectral and spatial data to FEM simulations (see Sec. S7), we are able to identify the low-*Q* mode. See Fig. S7.2 and S7.3 in the Supplementary Information [57] for the mode profiles of the high-*Q* and of the low-*Q* mode, respectively. Notably, the peak in the reflectivity spectrum associated with the Fabry–Pérot mode remains visible in this configuration, though the peak position is slightly shifted to 0.90 eV. This can be due to interferences with other modes in the InP membrane (see Sec. S7 in the Supplementary Information [57]) and due to geometrical differences of the *X* and *Y* axes of the sample.

**Figure 6:**
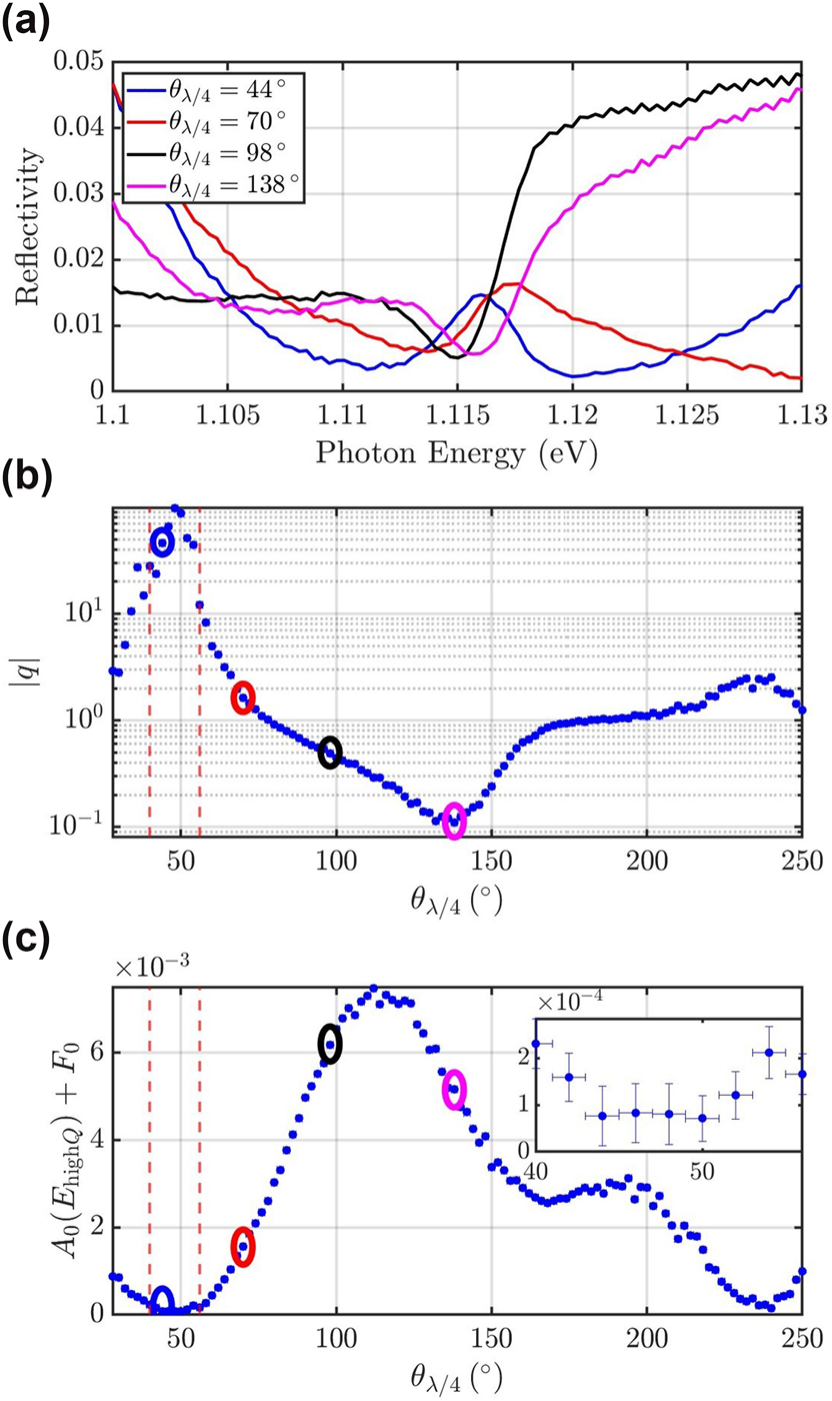
Lineshape as a function of *θ*_*λ*/4_. a) Spectra, b) extracted fit parameter |*q*| and c) extracted background *A*_0_(*E*_high*Q*_) + *F*_0_ for input-polarization $\hat{D}$ and detection polarization rotated by *θ*_*λ*__/2_ = −4° for various *θ*_*λ*__/4_ with respect to cross-polarization settings. The values for |*q*|, for the background, and for *θ*_*λ*/4_ marked by colored ellipses in b) and c) correspond to the spectra in a) with the same color. The red dashed lines indicate the angle range where the background reflectivity is $<3\times 10^{-4}$ while *q* > 12, also indicated as the inset in panel c).

Very importantly, we study the influence of polarization on the lineshape and on the background spectrum and find that the background can be fully suppressed for a finite range of frequencies by detecting a certain polarization. A *λ*/4 plate is now inserted in the detection path to suppress possible elliptically polarized states of the background. For the investigation, the input polarization is fixed to be along the $\hat{D}$ direction, while the *λ*/2 and *λ*/4 plates in the detection path are rotated by *θ*_*λ*/2_ and *θ*_*λ*/4_, respectively. All angles contain a measurement uncertainty of ±1°. Figure 6a depicts four representative spectra for various settings of *θ*_*λ*/4_ and for *θ*_*λ*/2_ = −4° in the spectral region of interest close to the high-*Q* mode. The angles *θ*_*λ*/2_ and *θ*_*λ*/4_ are defined with respect to the standard cross-polarization configuration, such that *θ*_*λ*/2_ = *θ*_*λ*/4_ = 0 corresponds to ${\hat{E}}_{\text{out}}=\hat{A}$ . Clearly, the spectra are transformed into a Lorentzian-like peak at *θ*_*λ*/4_ = 44°, see Figure 6a. As the polarization settings differ from simple parallel- or cross-polarization, the detection polarization differs from the optical axis of the beamsplitter and contains even elliptically polarized contributions. Therefore, the calibration of the beamsplitter presented in Sec. S3 is challenging, and a correction factor similar to *χ*(*ω*) as defined in Eq. S3.4 in the Supplementary Information [57] is difficult to obtain. Therefore, we disregard the correction factor *χ*(*ω*) and note that the reflectivity in Figure 6a might differ by about a factor of 1.5. We expect *χ*(*ω*) to be close to constant in the spectral range depicted in Figure 6a, so that the spectral shape is not influenced by *χ*(*ω*).

To further quantify the effect of the detection polarization on the lineshape, the *λ*/4 plate is rotated over a larger range while spectra are recorded. Each spectrum is fitted with Eq. (3). The parameters *E*_0_ and *γ* are fixed by the extracted parameters from the fit of the high-*Q* mode in parallel polarization (see Fig. A.1), leaving *F*_0_, *q*, and the offset spectrum as free fitting parameters.

Figure 6b shows the extracted parameter |*q*| and c) shows the extracted background reflectivity *A*_0_(*E*_high*Q*_) + *F*_0_, (see Sec. S1 in the Supplementary Information [57]) as a function of *θ*_*λ*/4_ for ${\hat{E}}_{\text{in}}=\hat{D}$ and for *θ*_*λ*/2_ = −4°. It can be noted that *q*, and in turn, the detected lineshape, depend crucially on *θ*_*λ*/4_. For *θ*_*λ*/4_ = 40° to 56°, |*q*| reaches values larger than 12. In this range, the background reflectivity reaches values $<3\times 10^{-4}$ , see Figure 6c. This shows that the background is almost fully suppressed, though the suppression only works in a narrow spectral range. This follows from the fact that the background $\vec{b}\left(E_{\text{ph}}\right)$ is energy-dependent (cf. Section 2). Therefore, the polarization of the background is a function of energy, and the background can only be suppressed in a finite range of frequencies. On the other hand, the background reflectivity reaches the maximal value of (7.48 ± 0.04) × 10^−3^ at *θ*_*λ*/4_ = 112°, which a factor of 25 higher than the smallest value of the background reflectivity. At this angle, |*q*| = 0.3, which is much smaller than the maximum value of |*q*| = 97.9 at *θ*_*λ*/4_ = 48°, showcasing the importance of *θ*_*λ*/4_ on the background suppression and the effectiveness of the approach. Since a Fano lineshape becomes a Lorentzian peak for *q* → ±*∞*, the value for *q* can be subject to a large uncertainty for *θ*_*λ*/4_ = 40° to 56°.

To quantify the error when approximating a Fano lineshape with a Lorentzian lineshape for finite |*q*|, we carry out an error analysis, see Sec. S1 in the Supplementary Information [57]. From Eq. S1.8, we find a normalized error of *ϵ*_norm_ < 0.0015 at *θ*_*λ*/4_ = 48°, highlighting that for this angle, the lineshape can be approximated by a Lorentzian function with a very small normalized error. In the indicated angle range of *θ*_*λ*/4_ = 40° to 56°, *ϵ*_norm_ is smaller than <0.09 . It is a main finding of this study that the background contributing to the Fano lineshape is polarized and that it is, therefore, possible to suppress the recorded background completely in a narrow spectral range while transforming a Fano lineshape into a Lorentzian lineshape by measuring in a specific polarization.

**Figure A.3:**
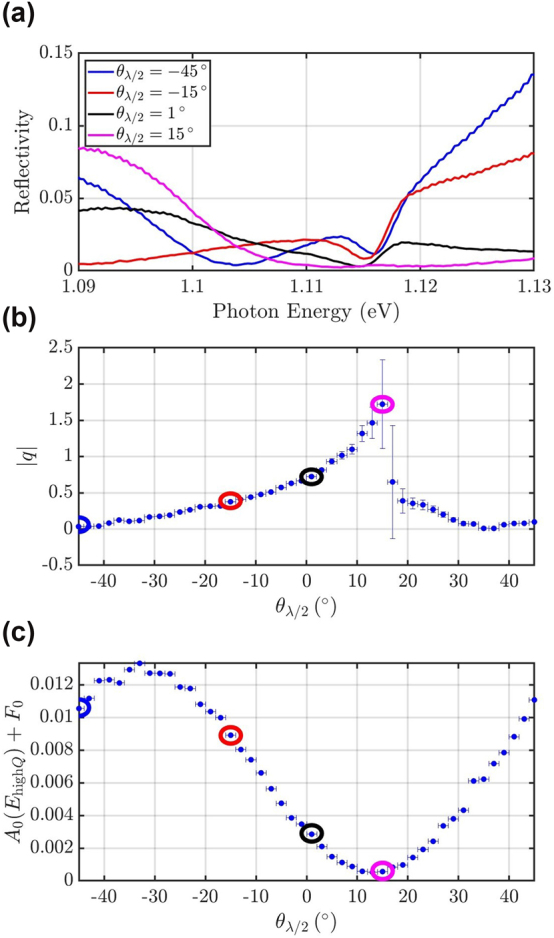
Lineshape as a function of *θ*_*λ*/2_. a) Spectra, b) extracted fit parameter |*q*| and c) extracted background *A*_0_(*E*_high*Q*_) + *F*_0_ for input-polarization $\hat{D}$ and detection polarization rotated by *θ*_*λ*/2_ with respect to cross-polarization settings. The values for |*q*|, for the background, and for *θ*_*λ*/2_ marked by colored ellipses in b) and c) correspond to the spectra in a) with the same colors.

In addition, we investigate the influence of the detection polarization on the lineshape without a *λ*/4 plate in appendix A.2 while optimizing *θ*_*λ*/2_. As apparent from Fig. A.3, the background suppression does not work as well as with the *λ*/4 plate. First of all, the background only reaches values $>5\times 10^{-4}$ , more than a factor of 1.6 larger than with the *λ*/4 plate. Furthermore, when the background reaches the smallest value, the spectral signal of the mode cannot be observed anymore, see *θ*_*λ*/2_ = 15° in Fig. A.3 a), which manifests itself in much smaller values for |*q*|. Without the *λ*/4 plate, |*q*| < 3, while with the *λ*/4 plate, |*q*| can get one order of magnitude larger.

**Figure 7:**
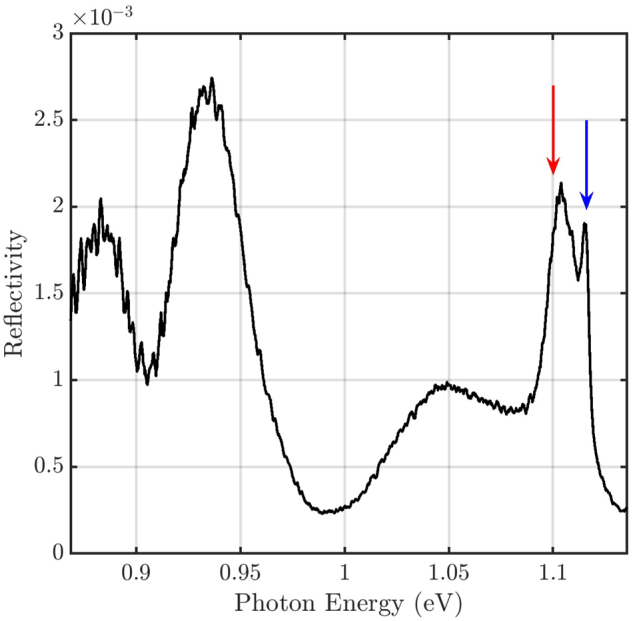
Spectrum in the center of the cavity for ${\hat{E}}_{\text{in}}=\hat{V}$ and ${\hat{E}}_{\text{out}}=\hat{H}$ . This configuration is symmetry-forbidden. The blue arrow marks the resonance energy of the high-*Q* mode, the red arrow of the low-*Q* mode (cf. Fig. A.1 and Fig. A.2).

Lastly, we turn our attention to spectra recorded with ${\hat{E}}_{\text{in}}=\hat{V}$ and ${\hat{E}}_{\text{out}}=\hat{H}$ . In this unusual cross-polarization setting, the input polarization is parallel to the high-*Q* mode but perpendicular to the low-*Q* mode, while the detection polarization is parallel to the low-*Q* mode but perpendicular to the high-*Q* mode. This polarization setting might be considered symmetry-forbidden because it specifically probes the *r*_*xy*_(*ω*) and *r*_*yx*_(*ω*) elements of the reflection matrix, which are forbidden by symmetry arguments (see Section 2). However, misalignment in the back focal plane and illumination off the center of the cavity can create non-zero off-diagonal elements. Figure 7 depicts a spectrum recorded in this configuration in the center of the cavity. The symmetry-forbidden nature is visible in the magnitude of the observed reflectivity, which is less than 0.3 % over the full spectral range. Surprisingly, both the high-*Q* mode and low-*Q* mode can still be observed. Other spectral features at lower eigenenergies are related to other modes in the InP membrane (see Fig. S7.1 in the Supplementary Information) and Fabry–Pérot modes forming in the air layer between the InP membrane and the silicon substrate as discussed above. A fit of the high-*Q* and the low-*Q* modes in Figure 7 with two Fano profiles simultaneously yields *q*_high*Q*_ = −9 ± 4 and *q*_low*Q*_ = 3 ± 1. These values are much smaller than with complete background suppression with a *λ*/4 plate (cf. Figure 6b), indicating that the symmetry-forbidden configuration does not necessarily suppress the background completely. This result demonstrates, however, that choosing these unusual cross-polarization settings allows us to resolve the modes simultaneously.

## 5 Concluding discussion

We investigated the spectral and spatial properties of an EDC cavity with polarization tomography. Firstly, we formulated a model for the electric field associated with a Fano lineshape intensity similar to the existing literature on this topic [39], [44], considering the vectorial properties of the individual contributions, namely the background and the high-*Q* mode. This vectorial description of the Fano lineshape is valid in a confocal geometry, though it is not limited to dielectric nanocavities and could be applied to other systems, such as plasmonic nanocavities.

We pursued reflection measurements in a confocal geometry and experimentally observed the previously reported high-*Q* mode of the EDC cavity, along with a low-*Q* mode with a lower quality factor, lower resonance energy, and orthogonal polarization compared to the high-*Q* mode. Moreover, we observe signatures of other modes in the InP membrane and Fabry–Pérot modes. We study the modes systematically with polarization tomography. Comparing our results to numerical calculations, we are able to identify the low-*Q* mode. Moreover, we demonstrated that the background, and in turn, the lineshape, crucially depend on the polarization. This very important finding allowed for complete suppression of the background for a certain pair of polarization angles. We showed that the lineshape can be transformed into a Lorentzian-like peak. Lastly, we showed that by choosing symmetry-forbidden cross-polarization settings, we can immediately retrieve information about several modes in the system. The presented results will be beneficial for the investigation and understanding of reflection measurements of dielectric nanocavities. The method has been proven effective for studying EDC cavities. Our approach relies on a cavity mode with well-defined polarization and a broad spectral background with a different polarization. Therefore, it extends beyond the studied structures and can be easily applied to other cavities, such as photonic-crystal cavities and plasmonic nanoparticles. We also highlight that the approach suggested in this paper could be extended to transmission measurements where it has been observed that cavity resonance lineshapes also depend critically on the polarization [39], [40].

## Supplementary Material

Supplementary Material Details

## A Appendix

### A.1 Fit of the lineshapes of the modes

Fig. A.1 depicts the reflected spectrum in parallel polarization ${\hat{E}}_{\text{in}}={\hat{E}}_{\text{out}}=\hat{V}$ aligned with the polarization of the high-*Q* mode at *X* = *Y* = *Z* = 0 (cf. Figure 5a). Moreover, a fit of the high-*Q* mode with a Fano function as per Eq. (3) is shown. The fit yields *E*_0,high*Q*_ = (1.1162 ± 0.0001) eV and *Q*_high*Q*_ = 265 ± 8, *F*_0,high*Q*_ = (14.3 ± 0.4) × 10^−3^ and *q*_high*Q*_ = 0.74 ± 0.02. The offset spectrum is determined as *A*_0_(*E*_ph_) ≈ − 0.91 + 0.82*E*_ph_/eV.

Fig. A.2 depicts the reflected spectrum in parallel-polarization ${\hat{E}}_{\text{in}}={\hat{E}}_{\text{out}}=\hat{H}$ aligned with the polarization of the low-*Q* mode at *X* = *Y* = *Z* = 0 (cf. Figure 5b). A fit of the low-*Q* mode with a Fano function after Eq. (3) yields *E*_0,low*Q*_ = (1.1007 ± 0.0003) eV and *Q*_low*Q*_ = 48 ± 1, *F*_0,low*Q*_ = (16.0 ± 0.3) × 10^−3^ and *q*_low*Q*_ = −0.45 ± 0.04. The offset spectrum is determined as *A*_0_(*E*_ph_) ≈ − 0.28 + 0.25*E*_ph_/eV.

### A.2 Polarization series without *λ*/4 plate

In addition to the investigation of the lineshape with a *λ*/4 plate, we present a quantitative analysis of the background suppression without the *λ*/4 plate, while optimizing the angle of the *λ*/2 plate. Fig. A.3 depicts spectra and extracted fit parameters from polarization-resolved measurements as a function of *θ*_*λ*/2_. In combination with the analyzer, we effectively rotate the detection polarization by 2*θ*_*λ*/2_. Fig. A.3 a) depicts representative reflection spectra for ${\hat{E}}_{\text{in}}=\hat{D}$ as a function of *θ*_*λ*/2_ and without a *λ*/4 plate. It can be seen that when the background vanishes around *θ*_*λ*/2_ = 15°, also the signal of the high-*Q* mode vanishes completely. The individual spectra recorded are fitted with Eq. (3), and the extracted fit parameter |*q*| is shown as a function of *θ*_*λ*/2_ in Fig. A.3 b). It is evident that |*q*| does not reach as large values as with the *λ*/4 plate (cf. Figure 6b). Moreover, the minimal value of the spectrum (given as *A*_0_ + *F*_0_, see Sec. S1 in the Supplementary Information) without the *λ*/4 plate is = (5.2 ± 0.3) × 10^−4^ for *θ*_*λ*/2_ = 13° (cf. Fig. A.3 c)). This value is larger than with the *λ*/4 plate ( $<3\times 10^{-4}$ over the angular range *θ*_*λ*/4_ = 40°–56°, see Figure 6c).
