# Supplementary material for: Confocal polarization tomography of dielectric nanocavities
Source: Nanophotonics. 2025 Apr 23;14(12):2161–71. doi: 10.1515/nanoph-2024-0744 (PMC12147554; doi:10.1515/nanoph-2024-0744)
Supplement: Supplementary file 1 — Supplementary Material Details [file j_nanoph-2024-0744_suppl_001.pdf]

Supplementary Information: Confocal polarization tomography of dielectric nanocavities

F. Schröder[1,2], M. P. van Exter[3], M. Xiong[1,2], G. Kountouris[1,2], M. Wubs[1,2], P. T. Kristensen[1,2], and N. Stenger[1,2]

[1]*Department of Electrical and Photonics Engineering, Technical University of Denmark, Ørsteds Plads 343, 2800 Kgs. Lyngby, Denmark*
[2]*NanoPhoton - Center for Nanophotonics, Technical University of Denmark, Ørsteds Plads 345A, 2800 Kgs. Lyngby, Denmark*
[3]*Huygens-Kamerlingh Onnes Laboratory, Leiden University, P.O. Box 9504, 2300 RA Leiden, The Netherlands*

S1 Explicit calculation of the intensity spectrum and error analysis

In this section, we explicitly show that the vector field in Eq. 2 is associated with a Fano-lineshape power spectrum (Eq. 3). We start from Eq. 2:

$$\vec{S}(x) = \vec{b} + \frac{\vec{a}}{1 - ix}\,, \tag{S1.1}$$

with $x = (\omega - \omega_0)/\gamma$. As described in Sec. 2, $\vec{b}$ depends on ω, whereas the frequency-dependence of $\vec{a}$ is neglected. Owing to the linearity of the system, we can choose the phase of the incoming light so that $\vec{a}$ is real. To calculate

the power spectrum produced by this field, we take the absolute square [1]:

$$
\begin{aligned}
P(x) &= |\vec{S}(x)|^2 = \left(\vec{b} + \frac{\vec{a}}{1-ix}\right) \cdot \left(\vec{b}^* + \frac{\vec{a}}{1+ix}\right) \\
&= |\vec{b}|^2 + \frac{|\vec{a}|^2}{1+x^2} + \frac{\vec{a}\cdot\vec{b}}{1+ix} + \frac{\vec{a}\cdot\vec{b}^*}{1-ix} \\
&= |\vec{b}|^2 + \frac{|\vec{a}|^2}{1+x^2} + \frac{\vec{a}\cdot\vec{b}(1-ix) + \vec{a}\cdot\vec{b}^*(1+ix)}{1+x^2} \\
&= |\vec{b}|^2 + \frac{|\vec{a}|^2}{1+x^2} + \frac{\vec{a}\cdot(\vec{b}+\vec{b}^*)}{1+x^2} + \frac{ix\vec{a}\cdot(\vec{b}^*-\vec{b})}{(1+x^2)} \\
&= |\vec{b}|^2 + \frac{|\vec{a}|^2 + 2\Re(\vec{a}\cdot\vec{b}) + x2\Im(\vec{a}\cdot\vec{b})}{1+x^2} \\
&= \tilde{B} + \frac{\tilde{A} + \tilde{C}x}{1+x^2}\,, \qquad \text{(S1.2)}
\end{aligned}
$$

where we have introduced

$$
\begin{aligned}
\tilde{A} &= |\vec{a}|^2 + 2\Re(\vec{a}\cdot\vec{b})\,, \\
\tilde{B} &= |\vec{b}|^2\,, \\
\tilde{C} &= 2\Im(\vec{a}\cdot\vec{b})\,, \qquad \text{(S1.3)}
\end{aligned}
$$

in wich $\Re()$ and $\Im()$ take the real and imaginary part of a complex number. Now, we can show that this is indeed the Fano lineshape from Eq. 3:

$$
\begin{aligned}
P(\omega) &= A_0 + F_0 \frac{(q + (\omega-\omega_0)/\gamma)^2}{1 + (\omega-\omega_0)^2/\gamma^2} \\
\Leftrightarrow P(x) &= A_0 + F_0 \frac{(q+x)^2}{1+x^2} \\
&= A_0 + F_0 + F_0 \frac{(q+x)^2}{1+x^2} - F_0 \frac{1+x^2}{1+x^2} \\
&= A_0 + F_0 + F_0 \frac{(q+x)^2 - (1+x^2)}{1+x^2} \\
&= A_0 + F_0 + F_0 \frac{(q^2-1) + 2qx}{1+x^2}\,, \qquad \text{(S1.4)}
\end{aligned}
$$

which is exactly the last line of Eq. S1.2 with

$$\begin{aligned}\tilde{A} &= F_0(q^2-1)\,, \\ \tilde{B} &= A_0+F_0\,, \\ \tilde{C} &= 2F_0q\,. \end{aligned} \tag{S1.5}$$

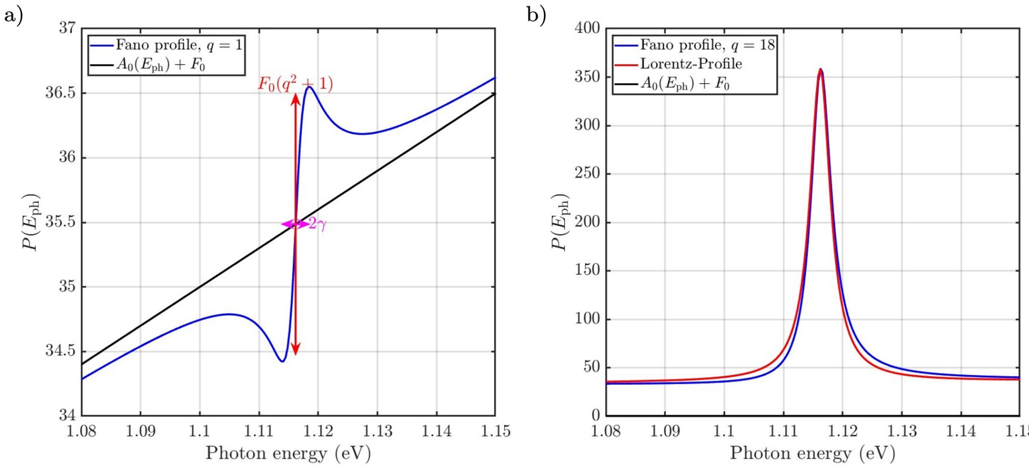

Figure S1.1: A Fano lineshape for $E_0 = 1.1162\,\text{eV}$, $\gamma = 2.1\,\text{meV}$, $F_0 = 1$, and $A_0(E_{\text{ph}}) = 1 + 30E_{\text{Ph}}/\text{eV}$ for **a)** $q = 1$ and **b)** $q = 18$. In b), a Lorentz-profile ($\tilde{C} = 0$) is plotted as a red line.

The parameter $A_0 = A_0(\omega)$ is a shorthand notation for the offset spectrum, and the spectral baseline, or background spectrum, is found to be A_0+F_0, see Fig. S1.1 a). The parameter F_0 relates to the amplitude via $F_0(q^2+1)$, see Fig. S1.1 a). The parameter q determines the overall shape and asymmetry of the spectrum.

With these equations, we can quantify the error when approximating a Fano lineshape with a Lorentzian function, e.g. approximating the blue curve with the red curve in Fig. S1.1 b). For that, we define a residual function r_q, given by the difference of a Fano lineshape P_{Fano} and a Lorentzian lineshape P_{Lorentz}, divided by the amplitude of the Lorentzian function. With Eqs. S1.2- S1.3

we find the residues:

$$
\begin{aligned}
r_q &= \frac{1}{\tilde{A}} \left(P_{\text{Fano}} - P_{\text{Lorentz}}\right) \\
&= \frac{1}{\tilde{A}} \left(\frac{\tilde{A} + \tilde{C}x}{1 + x^2} - \frac{\tilde{A}}{1 + x^2} \right) \\
&= \frac{\tilde{C}x}{\tilde{A}(1 + x^2)} = \frac{\tilde{C}(E_{\text{ph}} - E_0)/\gamma}{\tilde{A}(1 + ((E_{\text{ph}} - E_0)/\gamma)^2)} \, .
\end{aligned}
\tag{S1.6}
$$

Furthermore, we define an error function ϵ, given by integrating the squared residues over all frequencies

$$
\epsilon = \int_{-\infty}^{\infty} dE \, (r_q)^2 = \frac{\pi \tilde{C}^2 \gamma}{2\tilde{A}^2} \, . \tag{S1.7}
$$

Normalizing the error function with the decay rate yields $\epsilon_{\text{norm}} = \epsilon/\gamma$. Together with Eq. S1.5, we find:

$$
\epsilon_{\text{norm}} = \frac{\pi \tilde{C}^2}{2\tilde{A}^2} = \frac{2\pi q^2}{(q^2 - 1)^2} \, . \tag{S1.8}
$$

This can be interpreted as a normalized error function, stemming from approximating a Fano lineshape with a Lorentzian lineshape for finite q. To avoid dividing by zero, the whole calculation is only valid for $q \neq \pm 1 \Leftrightarrow \tilde{A} \neq 0$.

S2 Reference measurement on Au flake and longitudinal resolution

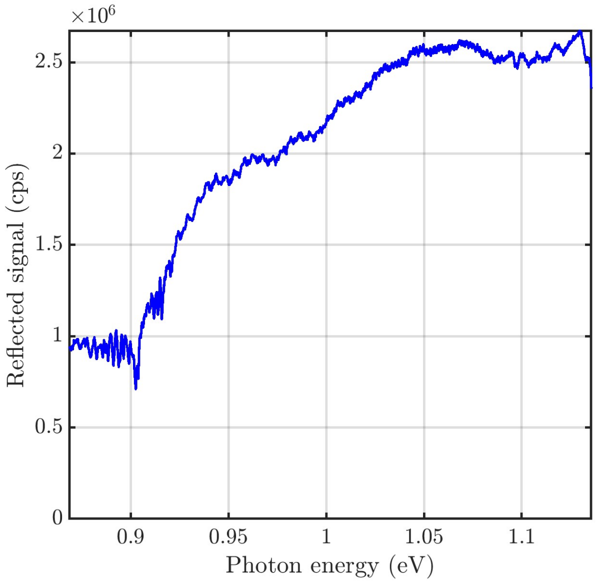

Figure S2.1: Typical reference spectrum on an Au flake in parallel polarization ($\hat{E}_{\text{in}} = \hat{E}_{\text{out}} = \hat{D}$).

A typical reflection spectrum on an Au flake to be used for normalization can be found in Fig. S2.1. Moreover, we measure the reflectivity of the reference gold flake as a function of the Z-position to estimate the out-of-plane resolution of the optical setup. Fig. S2.2 shows the reflectivity at the photon energy of interest $E_{\text{ph}} = 1.116\,\text{eV}$, which is the resonance energy of the high-Q cavity mode (see Sec. A.1). Fitting the reflectivity with a Gaussian function yields an out-of-plane FWHM of $(2.0 \pm 0.1)\,\mu\text{m}$. The focus and the focal depth can be slightly wavelength-dependent due to chromatic aberration.

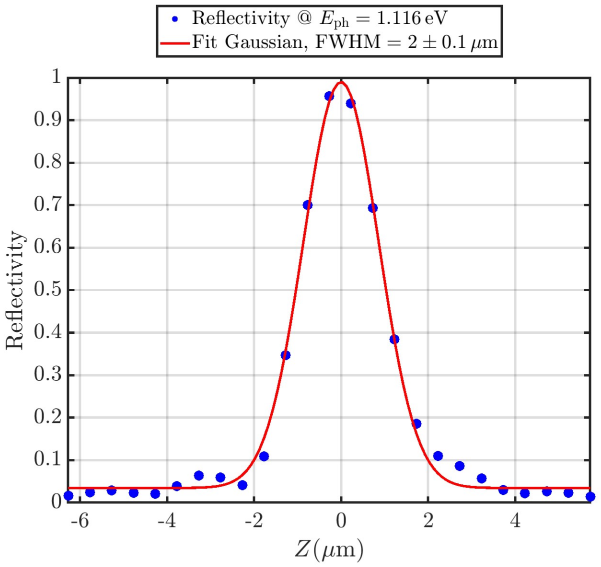

Figure S2.2: Reflectivity of a gold flake used for reference at $E_{ph} = 1.116\,\text{eV}$ as a function of the Z-position in parallel polarization ($\hat{E}_{in} = \hat{E}_{out} = \hat{D}$).

S3 Calibration of setup and beamsplitter

All reflection spectra in the main text are presented as reflectivities. We obtain these data by comparing the reflection spectrum of our nanocavity sample with that of a gold flake, which acts as a close-to-perfectly-reflecting reference. Hence, we determine the reflectivity $\tilde{R}$ as

$$\tilde{R}(\omega) = \frac{R_{\text{sam}}(\omega)}{R_{\text{ref}}(\omega)}\,, \tag{S3.1}$$

where R_{sam} and R_{ref} denote the reflection coefficient of the sample and of the reference, respectively. This calibration works fine for measurements with parallel polarization but needs a correction in crossed polarization with an unbalanced beamsplitter. We will denote the reflection and transmission coefficients of the beamsplitter as R_s, R_p, T_s and T_p for s- and p-polarized light. Measurements on the gold flake with identical input and output polarizations gave almost identical results for s and p polarizations and thereby showed that $R_sT_s = R_pT_p$. Hence, we only need to introduce a single correction function

$$\chi(\omega) \equiv \frac{R_s(\omega)}{R_p(\omega)} = \frac{T_p(\omega)}{T_s(\omega)}\,. \tag{S3.2}$$

Consider a setup where the input is p-polarized and the detection is s-polarized and compare the measured reflection spectrum $R_{\text{sam},ps}(\omega)$ with the $R_{\text{ref},pp}(\omega)$ reference spectrum obtained with p-polarized input and output. The ratio of these spectra yields the measured reflectivity

$$R_{\text{meas.},ps}(\omega) = \frac{R_{\text{sam},ps}}{R_{\text{ref},pp}} = \frac{R_s}{R_p}\tilde{R}_{ps}(\omega) = \chi(\omega)\tilde{R}_{ps}(\omega)\,, \tag{S3.3}$$

where $\tilde{R}_{ps}(\omega)$ is the reflectivity. A similar calculation for s-polarized input and p-polarized output yields $R_{\text{meas.},sp}(\omega) = \tilde{R}_{sp}(\omega)/\chi(\omega)$. To determine $\chi(\omega)$, we divide these two equations, using $\tilde{R}_{ps}(\omega) = \tilde{R}_{sp}(\omega)$ from reciprocity, to find:

$$\chi(\omega) = \sqrt{\frac{\tilde{R}_{\text{meas.},ps}}{\tilde{R}_{\text{meas.},sp}}}\,. \tag{S3.4}$$

These calculations only work if the input- and output light are aligned with the s and p axes of the beamsplitter. We therefore chose to record the $\hat{D}$ and $\hat{A}$ polarizations (cf. Fig 1) with the sample being rotated by 45°. For more general polarizations, we would need to include a potential phase shift between s- and p-polarized light throughout the setup.

S4 Lateral resolution of the setup

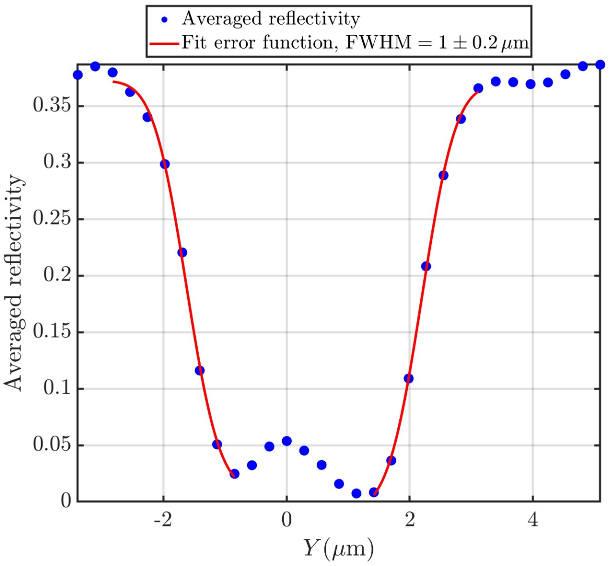

Figure S4.1: Averaged reflectivity of the data presented in Fig. 3 as a function of the Y-position and for $X = Z = 0$ in parallel polarization ($\hat{E}_{\text{in}} = \hat{E}_{\text{out}} = \hat{D}$).

To estimate the lateral resolution of the setup, we analyze the position scan of the cavity in parallel configuration. Fig. 3 in the main text depicts the reflection spectra as a function of Y with $\hat{E}_{\text{in}} = \hat{E}_{\text{out}} = \hat{D}$. The reflectivity for each spectrum is averaged over the spectral range of the measurements ($0.87 - 1.13\,\text{eV}$) and plotted as a function of Y in Fig. S4.1. We fit the edges of the averaged reflectivity R_{av} with an error function erf(x), resulting from a convolution of a Gaussian function with a step function [2]:

$$R_{\text{av}} = R_{\text{off}} + A\,\text{erf}\left(\frac{E_{\text{ph}} - E_c}{\sqrt{2}\sigma}\right), \tag{S4.1}$$

with R_{off} denoting an overall offset in the reflectivity, A denoting an amplitude, E_c denoting the center position of the Gaussian, and σ denoting the standard deviation of the Gaussian from which the FWHM is calculated as $\text{FWHM} = 2\sqrt{2\ln(2)}\,\sigma$. The error function is given as [2]:

$$\text{erf(x)} = \frac{2}{\pi}\int_0^x dt\, e^{-t^2} \tag{S4.2}$$

From the fit in Fig. S4.1, a FWHM of $(1.0 \pm 0.2)\,\mu\text{m}$ is determined.

For a diffraction-limited spot, the intensity in the focal plane is $\propto (2J_1(x)/x)^2$, with $x = \frac{2\pi}{\lambda} NA\,\rho$, where NA denotes the numerical aperture (= 0.65 for our 50× objective) of the objective and ρ denotes the lateral displacement [3]. This function has its first zero-value at $\rho = 0.61\frac{\lambda}{NA}$, which is often referred to as the diffraction limit. The FWHM of this function is given by $\approx 0.51\frac{\lambda}{NA} = 0.87\,\mu\text{m}$ at the photon energy of interest $E_{\text{ph}} = 1.116\,\text{eV}$ (the resonance energy of the high-Q cavity mode).

S5 Far-field position scans

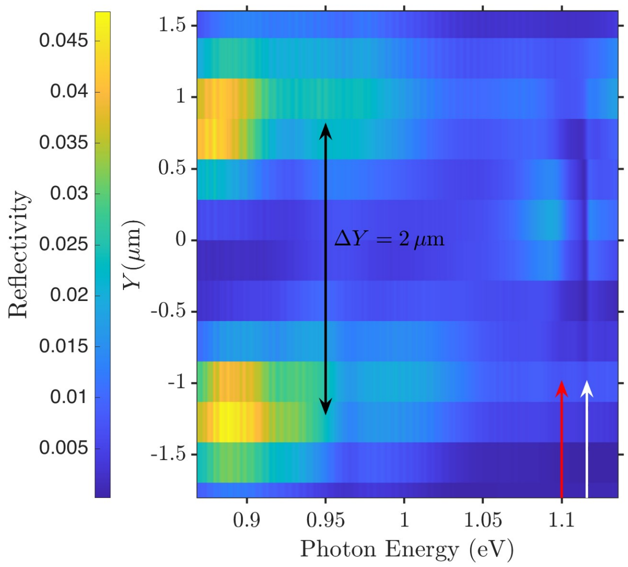

Figure S5.1: Reflection spectra as a function of the Y position and for $X = Z = 0$ in the conventional cross-polarization configuration $\hat{E}_{\mathrm{in}} = \hat{A}$, $\hat{E}_{\mathrm{out}} = \hat{D}$. The high-$Q$ and low-Q modes are marked with white and red arrows, respectively. The black arrow depicts the spatial distance of the maxima, related to reflection from the outer ring of the cavity.

Fig. S5.1 depicts the full scan of the Y position in cross-polarization ($\hat{E}_{\mathrm{in}} = \hat{A}$, $\hat{E}_{\mathrm{out}} = \hat{D}$) of the spectra discussed in Fig. 4. It can be seen that the high-Q mode and low-Q mode, marked with white and red arrows, respectively, are visible in the center of the cavity. The reflectivity maxima are separated by $\approx 2.0\,\mu\mathrm{m}$, marked by the black arrow, which fits well with the diameter of the outer rings of the cavity.

Fig. S5.2 depicts the reflected signal as a function of the Y-position with the input and output polarization aligned with the high-Q mode (see main text). The high-Q mode is clearly located in the center of the cavity. For further demonstration, we depict the extracted fit parameter for the amplitude $F_0(q^2 + 1)$ for two position scans along the X and the Y axis, cf. Fig. S5.3. For the various fits, the parameters E_0 and γ were fixed to the values extracted from the fit in the center of the cavity, see Fig. A.1. The mode am-

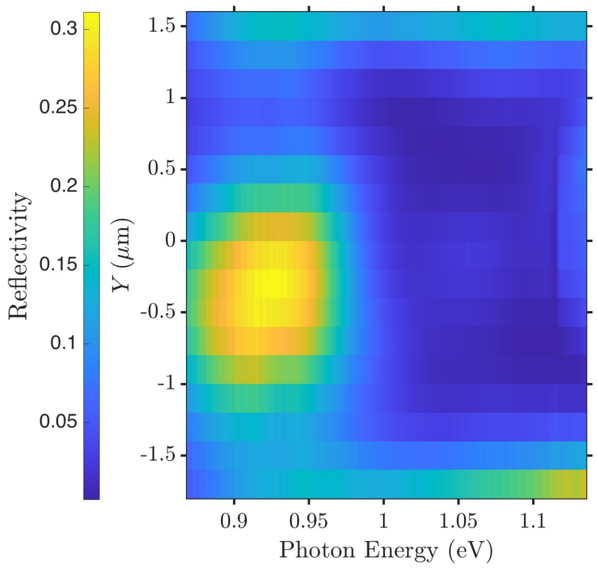

Figure S5.2: Reflection spectra of the cavity as a function of the Y-position and for $X = Z = 0$ in parallel polarization aligned with the high-Q mode ($\hat{E}_{\text{in}} = \hat{E}_{\text{out}} = \hat{V}$).

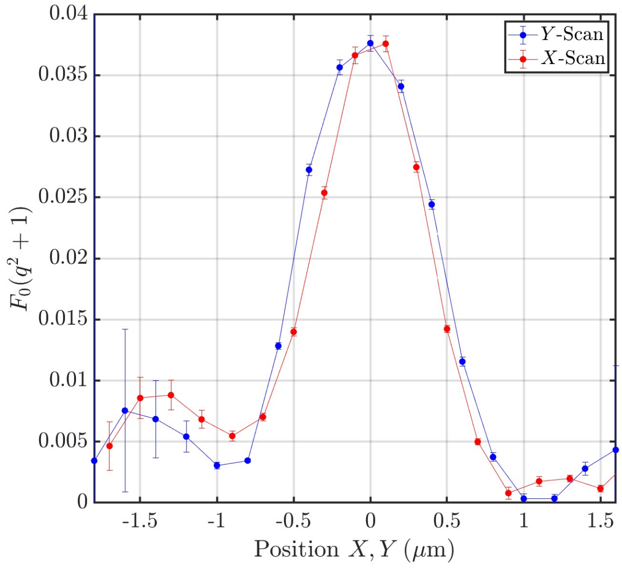

Figure S5.3: Extracted fit parameter $F_0(q^2+1)$ of the high-Q mode for two spatial scans of the X and the Y positions, respectively (cf. Fig. S5.2). Lines are guides to the eye.

plitude is clearly centered in the cavity's center with a FWHM of $\approx 1.0\,\mu m$, which is exactly the extracted lateral resolution of the setup (see Sec. S4).

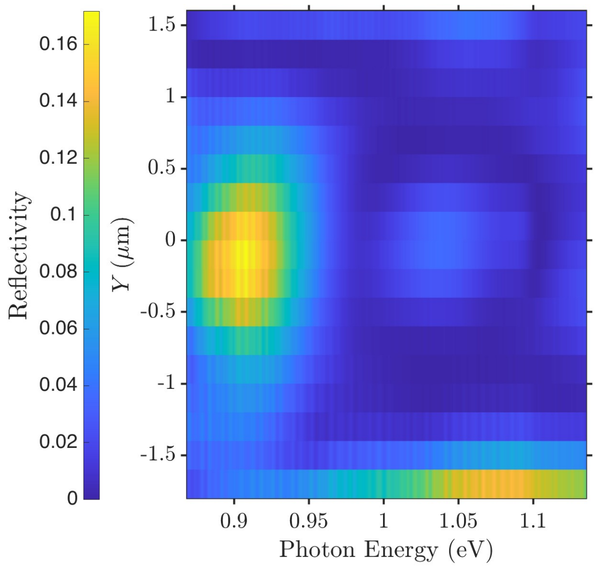

Figure S5.4: Reflection spectra of the cavity as a function of the Y-position and for $X = Z = 0$ in parallel polarization aligned with the low-Q mode ($\hat{E}_{\text{in}} = \hat{E}_{\text{out}} = \hat{H}$).

To investigate spatial properties of the low-Q mode, position scans with $\hat{E}_{\text{in}} = \hat{H}$, $\hat{E}_{\text{out}} = \hat{H}$ are pursued. Fig. S5.4 depicts exemplary reflectivity as a function of the Y-position. The fit parameter $F_0(q^2+1)$ for the low-Q mode as a function of the X and the Y positions is presented in Fig. S5.5. Here, the values for E_0 and for γ_0 were fixed as well. The low-Q mode seems to be less confined than the high-Q mode. In the X direction, $F_0(q^2+1)(X)$ has a FWHM of $\approx 1.8\,\mu m$, compared to $\approx 1.0\,\mu m$ as for the high-Q mode. In the Y direction, the central part of the mode is sharply peak but $F_0(q^2+1)(Y)$ increases again for larger Y values, which shows that the mode is even less confined in this direction. The reason for the asymmetry in the Y direction is most likely a slight misalignment of the sample with respect to the symmetry axes of the cavity.

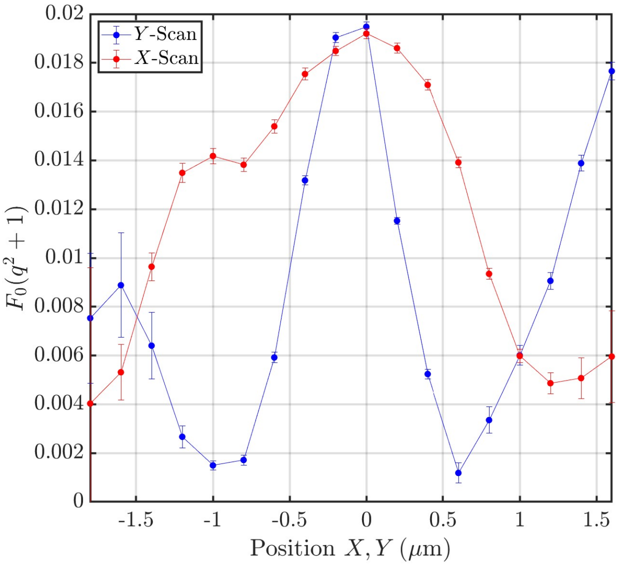

Figure S5.5: Extracted fit parameter $F_0(q^2 + 1)$ of the low-Q mode for two spatial scans of the X and the Y positions, respectively (cf. Fig. S5.4). Lines are guides to the eye.

S6 Near-field measurements

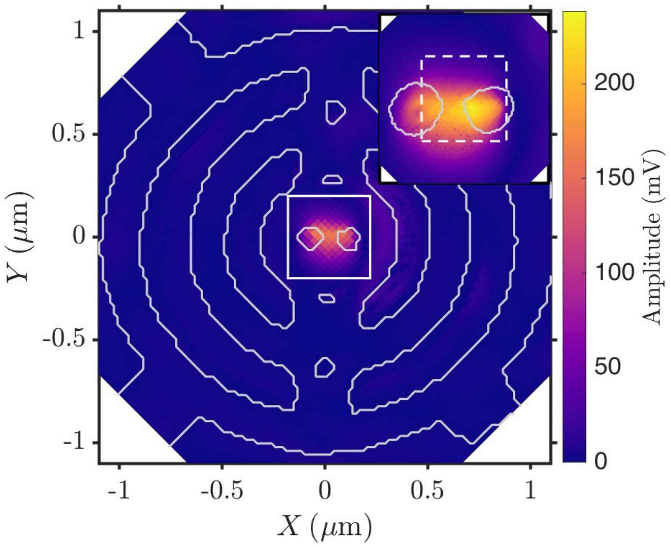

Figure S6.1: s-SNOM measurements of the 3rd-order scattering amplitude. The input and output polarizations are both along the $\hat{V}$ direction, aligned with the high-Q mode [4, 5]. The photon energy of a tunable laser is 1.105 eV, aligned with the resonance of the high-Q mode in the s-SNOM setup, cf. Fig. S6.2. Solid contour lines depict the AFM profile of the EDC cavity. The inset shows a high-resolution scan of the center region, while the dashed rectangle in the center of the inset depicts the spatial window for averaging of the amplitude to retrieve the spectrum.

Scattering-type scanning near-field optical microscope (s-SNOM) measurements [6] are often carried out to investigate the electric-field distribution in dielectric nanocavities [4, 5]. In a s-SNOM, the apex of an atomic force microscope (AFM) tip, operating in tapping mode, is illuminated by a tunable laser [7]. The detected scattered field is demodulated at higher harmonic orders M of the tip-tapping frequency. Demodulation at $M \geq 3$ allows for retrieval of the near-field signal [8]. We present data demodulated at the 3rd harmonic order. Furthermore, pseudo-heterodyne detection [9] is exploited to increase the near-field signal strength while suppressing the far-field signal.

We pursue s-SNOM measurements of the cavity with different polarizations and wavelengths. This way, we can retrieve the electric-field distribution of the high-Q mode. Fig. S6.1 depicts the 3rd-order scattering amplitude of

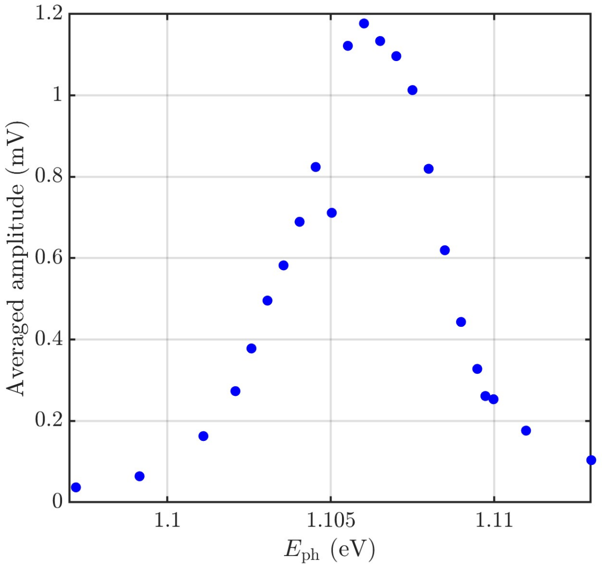

Figure S6.2: Average of 3rd-order scattering amplitude in a spatial window of 200 nm x 200 nm, cf. Fig. S6.1. The input and output polarizations are both aligned with the $\hat{V}$ direction to detect the high-Q mode.

the cavity as a function of X and Y. Here, the wavelength of a tunable laser is in resonance with the high-Q cavity mode, see also Fig. S6.2. As in previous studies [4, 5], the input and output polarizations are aligned in-plane and parallel to the high-Q mode. Fig. S6.2 depicts the averaged 3rd-order scattering amplitude in a spatial window of 200 nm x 200 nm in the center of the cavity. Clearly, a resonant behavior can be observed. The shift of $\approx$ 10 meV of the resonance energy compared to far-field measurements (see Fig. A.1) can be explained by the influence of the AFM tip on the cavity resonance.

Efficient near-field measurement of the low-Q mode is not possible as the low-Q mode is, to a large extent, located in a void region (see Fig. S7.3). Thus, if the tip enters such a void region, the excitation laser light is shadowed by the surrounding dielectric material, so that solely scattering from the shaft of the AFM needle remains [4, 5].

S7 FEM simulations

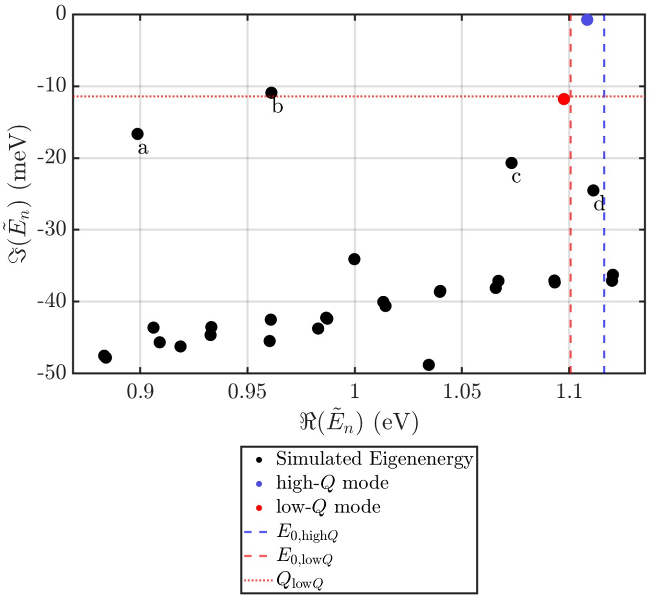

Figure S7.1: Complex eigenenergies $\tilde{E}_n$. The blue and red markers denote the simulated eigenenergy of the high-Q and low-Q modes, respectively. The blue- and the red-dashed lines mark the measured resonance energies of the high-Q and of the low-Q modes (cf. Sec. A.1), respectively. The red-dotted line marks the decay rate corresponding to the Q-factor of the low-Q mode. Modes corresponding to other spectral features in Fig. 4 and Fig. 7 are labeled as a-d.

The spectral and spatial information from the experiment are compared to finite-element-method simulation [10]. We numerically solve the Helmholtz equation

$$\nabla \times \nabla \times \tilde{\mathbf{f}}_n(\mathbf{r}) - \tilde{k}_n^2 \epsilon_{\mathrm{R}}(\mathbf{r}) \tilde{\mathbf{f}}_n(\mathbf{r}) = 0 \qquad \text{(S7.1)}$$

with scattering boundary conditions. $\tilde{\mathbf{f}}_n(\mathbf{r})$ denotes the electric field of the eigenmode, $\tilde{k}_n^2 = \tilde{\omega}_n/c$ the corresponding wavenumber with the eigenfrequency $\tilde{\omega}_n$ and $\epsilon_{\mathrm{R}}(\mathbf{r})$ the relative permittivity. This way, we retrieve the eigenmodes of the system in the quasinormal mode framework [11]. We restrict our calculation to the InP membrane surrounded by air, and apply sufficient symmetry conditions for the high-Q and the low-Q mode, respectively (see below). As the sample exhibits mirror symmetry with respect to the X and the Y axes, the electric field distribution of every mode must be

symmetric to either a perfect electric conductor (PEC) or a perfect magnetic conductor (PMC) boundary conditions along the symmetry axes.

Fig. S7.1 shows the simulated eigenenergies $\tilde{E}_n = \hbar\tilde{\omega}_n$. The values for the high-Q mode ($\tilde{E}_{\mathrm{high}Q}$) and the low-$Q$ mode ($\tilde{E}_{\mathrm{low}Q}$) have been calculated with a convergence study as described in Ref. [10]. Those values are indicated with blue and red markers and arrows, respectively. The calculated real part of the eigenenergies is red-detuned from the value observed experimentally, indicated by the blue and red dashed lines, for both modes. This can be explained by fabrication imperfections. As described in Ref. [10], the resonance energy is very sensitive to slight variations in the geometry. We find that decreasing the radius of the holes in the center of the cavity by 5 nm decreases the real part of the resonance energy of the high-Q mode by approximately 30 meV, see below. That is why we adjust the nominal geometry according to the SEM image (Fig. 1). As the cavity depicted in Fig. 1 is a clone of the studied cavity, small variations of the geometry might still appear. Moreover, surface roughnesses and oxidation effects can influence the resonance energies.

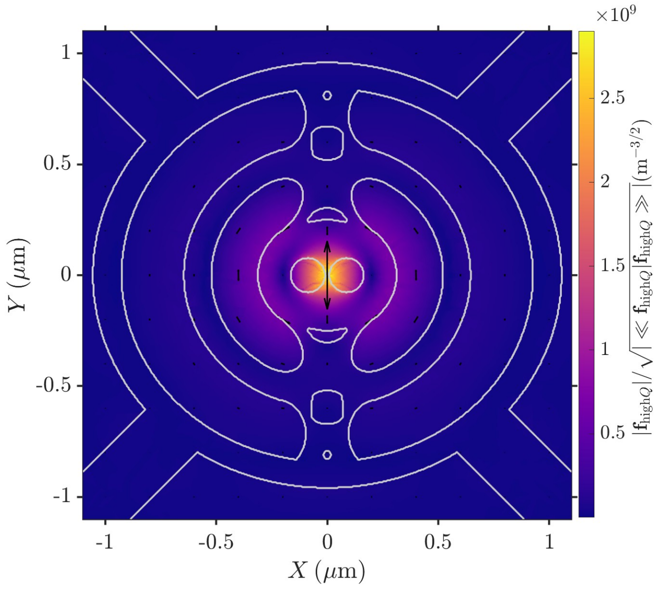

Figure S7.2: Normalized field profile of the high-Q mode, evaluated in a plane 25 nm above the EDC cavity. The white line draws the contour of the EDC cavity. The black arrows denote the in-plane orientation of the electric field.

We find $\Re(\tilde{E}_{\mathrm{high}Q}) = (1.10834 \pm 1 \times 10^{-5})$ eV, which is around 8 meV smaller than the experimental value $E_{0,\mathrm{high}Q} = (1.1162 \pm 0.0001)$ eV, cf. Fig. A.1. The calculated Q-factor of the high-Q mode is 719 ± 3, which is much larger than the measured $Q_{\mathrm{high}Q} = 265 \pm 8$, a discrepancy which is very common in dielectric nanocavities [4, 5]. The simulated resonance energy of the low-Q mode $\Re(\tilde{E}_{\mathrm{low}Q}) = (1.0975 \pm 0.0002)$ eV differs by 3 meV from the measured value $E_{0,\mathrm{low}Q} = (1.1007 \pm 0.0003)$ eV. Moreover, the calculated Q factor for the low-Q mode is 46.6 ± 0.4. This value agrees well with the measured value $Q_{\mathrm{low}Q} = 48 \pm 1$, indicated by the dotted line in Fig. S7.1. We emphasize that the presented uncertainty is only the numerical uncertainty for a given structure. Fabrication of dielectric nanocavities is challenging, and shape variations on the nanometer scale are likely to occur. To estimate the uncertainty induced by geometrical variations, we study their influence on the complex eigenenergy below.

The normalized field profile $|\mathbf{f}_{\mathrm{high}Q}|/\sqrt{|\langle\langle \mathbf{f}_{\mathrm{high}Q}|\mathbf{f}_{\mathrm{high}Q}\rangle\rangle|}$ evaluated in a plane 25 nm above the EDC cavity corresponding, to the effective scattering distance between of the s-SNOM tip and the sample surface [5], is shown in Fig. S7.2. The mode is located in the center of the EDC cavity, which is verified with polarization tomography (Fig. S5.3) and near-field measurements, see Sec. S6. The in-plane orientation of the electric field demonstrates polarization mainly along the $\hat{V}$ direction, matching experimental observations, see Sec. 4. The high-Q mode is symmetric with a PEC boundary condition on the XZ-plane and a PMC boundary condition on the YZ-plane, as well as a PMC layer on the XY-plane [10].

The normalized field profile $|\mathbf{f}_{\mathrm{low}Q}|/\sqrt{|\langle\langle \mathbf{f}_{\mathrm{low}Q}|\mathbf{f}_{\mathrm{low}Q}\rangle\rangle|}$ evaluated in a plane 25 nm above the EDC cavity is shown in Fig. S7.3. The mode is extended in the X- and Y-directions, matching polarization tomography measurements, see Fig. S5.5. From the in-plane orientation of the electric field, it follows that the mode is mainly polarized along the $\hat{H}$ direction, matching experimental observations, see Sec. 4. The low-Q mode is symmetric with a PMC layer on the XZ-plane and a PEC layer on the YZ-plane, as well as a PMC layer on the XY-plane.

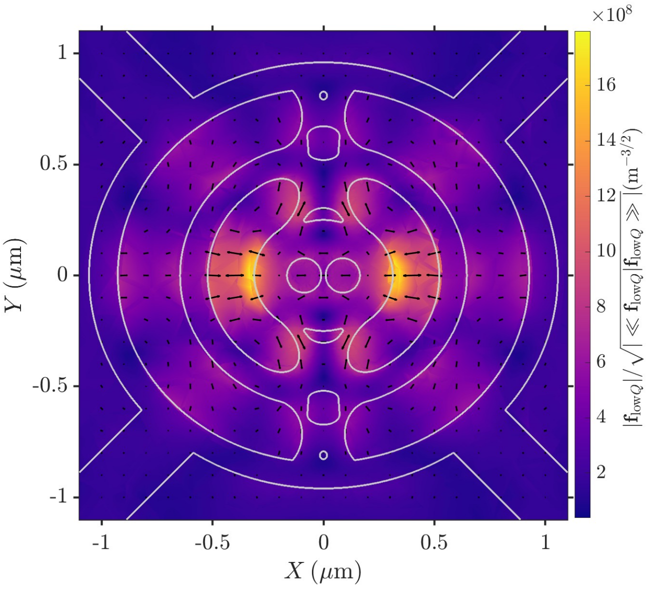

Figure S7.3: Normalized field profile of the low-Q mode, evaluated in a plane 25 nm above the EDC cavity. The white line draws the contour of the EDC cavity. The black arrows denote the in-plane orientation of the electric field.

To compare the simulation results with the experiments, we summarize the resonance energies and Q factors of the studied modes in Tab. 1, highlighting good agreement between experimental and simulation results.

**Simulation**	high-Q	low-Q		**Experiments**	high-Q	low-Q
$\Re(\tilde{E}_n)$ (eV)	1.108	1.098		E_0 (eV)	1.116	1.101
Q_{sim}	719 ± 3	47		Q	265 ± 8	48 ± 1

Table 1: Simulated and experimental values of the eigenenergy of the high-Q and the low-Q modes.

Fig. 4 and Fig. 7 exhibit Fano-shaped spectral features next to the studied high-Q and low-Q modes. These can be explained by more modes in the InP membrane, together with Fabry-Pérot modes forming between the membrane and the silicon substrate. From the FEM simulations, we find that there exist more modes with moderate Q factor in the InP membrane, labeled as a-d in Fig. S7.1. In addition, there can be Fabry-Pérot-type modes, as discussed in Sec. 4 in the main text. These are not captured in the eigenmode simulation, as the simulation domain is restricted to the InP membrane and

does not include the silicon substrate. Nevertheless, these Fabry-Pérot modes are clearly visible in the experimental data; see, for example, Fig. 5 in the main text, and can be explained by a simple model, as described in the main text. Also, we emphasize that these values are likely to shift slightly due to variations in the geometry due to imperfect fabrication, which we systematically study below.

Estimating the uncertainty introduced by geometrical variations can be effectively achieved with perturbation theory and quasinormal modes [10]. Alternatively, the geometry can be manually adjusted, followed by a recalculation of the eigenenergy. We choose the latter approach, and focus on varying the radius of the inner holes and the opening of the outer void regions as indicated in Fig. S7.4 a).

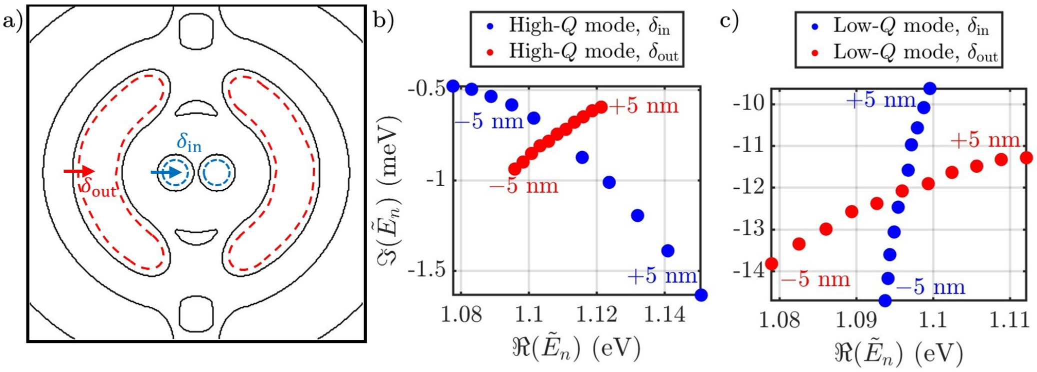

Figure S7.4: Shape deformations of the structure and their influence on the complex eigenenergies. a) Sketch of shape deformations studied, b) influence on the high-Q mode, and c) influence on the low-Q mode.

Panel b) depicts the complex eigenenergy of the high-Q mode as a function of δ_{in} and δ_{out}. For $\delta_{\text{in}} = -5\,\text{nm}$, we find $\Re(\tilde{E}_{\text{high}Q}) = 1.078\,\text{eV}$, and for $\delta_{\text{in}} = 5\,\text{nm}$, we find $\Re(\tilde{E}_{\text{high}Q}) = 1.151\,\text{eV}$. The complex part shifts from $\Im(\tilde{E}_{\text{high}Q}) = -0.5\,\text{meV}$ to $\Im(\tilde{E}_{\text{high}Q}) = -1.6\,\text{meV}$ for $\delta_{\text{in}} = -5\,\text{nm}$ and $\delta_{\text{in}} = 5\,\text{nm}$, respectively. The variation of the high-Q mode is larger against δ_{in} than against δ_{out}. Varying δ_{out} from -5 to $5\,\text{nm}$, we find $\tilde{E}_{\text{high}Q} = (1.096 - i\,0.9 \times 10^{-3})$ eV to $\tilde{E}_{\text{high}Q} = (1.121 - i\,0.6 \times 10^{-3})$ eV. Panel c) depicts the variation of the eigenfrequency against geometrical variations of the low-Q mode. Varying δ_{in} from -5 to $5\,\text{nm}$, we find $\tilde{E}_{\text{low}Q} = (1.094 - i\,14.7 \times 10^{-3})$ eV to $\tilde{E}_{\text{low}Q} = (1.099 - i\,9.6 \times 10^{-3})$ eV. Moreover, varying δ_{out} from -5 to $5\,\text{nm}$,

we find $\tilde{E}_{\mathrm{low}Q} = (1.079 - i\,13.8 \times 10^{-3})$ eV to $\tilde{E}_{\mathrm{low}Q} = (1.112 - i\,11.3 \times 10^{-3})$ eV.

In conclusion, the induced uncertainty by geometrical shape deformations is much larger than the numerical uncertainty for a given structure.

References

[1] P. T. Kristensen, J. R. De Lasson, M. Heuck, N. Gregersen, and J. Mork, “On the Theory of Coupled Modes in Optical Cavity-Waveguide Structures,” *J. Light. Technol.*, vol. 35, no. 19, pp. 4247–4259, 2017, DOI: 10.1109/JLT.2017.2714263.

[2] L. C. Andrews, *Special Functions of Mathematics for Engineers*, 2nd. SPIE—The International Society for Optical Engineering and Oxford University Press, 1997, DOI: 10.1117/3.270709.

[3] B. E. A. Saleh and M. C. Teich, *Fundamentals of photonics*. New York: Wiley, 1991, xviii, 966 H1 – Library of Congress H2 –TA1520.

[4] M. Albrechtsen, B. Vosoughi Lahijani, R. E. Christiansen, *et al.*, “Nanometer-scale photon confinement in topology-optimized dielectric cavities,” *Nat. Commun.*, vol. 13, no. 1, p. 6281, 2022, DOI: 10.1038/s41467-022-33874-w.

[5] M. Xiong, R. E. Christiansen, F. Schröder, *et al.*, “Experimental realization of deep sub-wavelength confinement of light in a topology-optimized InP nanocavity,” *Opt. Mater. Express*, vol. 14, no. 2, p. 397, 2024, DOI: 10.1364/OME.513625.

[6] M. B. Raschke and C. Lienau, “Apertureless near-field optical microscopy: Tip-sample coupling in elastic light scattering,” *Appl. Phys. Lett.*, vol. 83, no. 24, pp. 5089–5091, 2003, DOI: 10.1063/1.1632023.

[7] L. N. Casses, B. Zhou, Q. Lin, *et al.*, “Full Quantitative Near-Field Characterization of Strongly Coupled Exciton–Plasmon Polaritons in Thin-Layered WSe 2 on a Monocrystalline Gold Platelet,” *ACS Photonics*, vol. 11, no. 9, pp. 3593–3601, 2024, DOI: 10.1021/acsphotonics.4c00580.

[8] B. Knoll and F. Keilmann, “Enhanced dielectric contrast in scattering-type scanning near-field optical microscopy,” *Opt. Commun.*, vol. 182, no. 4, pp. 321–328, 2000, DOI: https://doi.org/10.1016/S0030-4018(00)00826-9.

[9] N. Ocelic, A. Huber, and R. Hillenbrand, “Pseudoheterodyne detection for background-free near-field spectroscopy,” *Appl. Phys. Lett.*, vol. 89, no. 10, pp. 87–90, 2006, DOI: 10.1063/1.2348781.

[10] G. Kountouris, J. Mørk, E. V. Denning, and P. T. Kristensen, "Modal properties of dielectric bowtie cavities with deep sub-wavelength confinement," *Opt. Express*, vol. 30, no. 22, p. 40 367, 2022, DOI: 10.1364/OE.472793.

[11] P. Trøst Kristensen, K. Herrmann, F. Intravaia, and K. Busch, "Modeling electromagnetic resonators using quasinormal modes: Erratum," *Adv. Opt. Photonics*, vol. 13, no. 4, p. 834, 2021, DOI: 10.1364/aop.446675.
